# *Aloe vera* (L.) Webb.: Natural Sources of Antioxidants – A Review

**DOI:** 10.1007/s11130-019-00747-5

**Published:** 2019-06-18

**Authors:** Marzanna Hęś, Krzysztof Dziedzic, Danuta Górecka, Anna Jędrusek-Golińska, Elżbieta Gujska

**Affiliations:** 1 Faculty of Food Science and Nutrition, Poznań University of Life Sciences, Wojska Polskiego 31, 60-624 Poznań, Poland; 2 Department of Pediatric Gastroenterology and Metabolic Diseases, Poznan University of Medical Sciences, Szpitalna 27/33, 60-572 Poznań, Poland; 3 Department of Commodity Sciences and Food Analysis, University of Warmia and Mazury in Olsztyn, Plac Cieszyński 1, 10-957 Olsztyn, Poland

**Keywords:** Natural antioxidants, Bioactive components, Antioxidative activity, Aloe vera

## Abstract

Many studies have proved that bioactive components of *Aloe vera* have an anti-inflammatory effect and support lipid and carbohydrate metabolism, helping to maintain normal sugar and cholesterol levels in blood and normal body weight. When aloe is applied externally, it accelerates the regeneration of the damaged skin. Aloe contains antioxidants, which may increase the shelf-life and nutritional value of food; therefore, it is widely used in cosmetic, pharmaceutical and food industry. An antioxidant activity was shown for leaf’s skin, flowers and gel of aloe. In this work the future of *A. vera* as effective antioxidants is primarily discussed and expected trends are summarised. Furthermore, the bioactive components and the health-promoting effects of *A. vera* are investigated.

## Introduction

Antioxidants are substances that prevent oxidation of other compounds. To prevent food degradation due to oxidation, employment of antioxidants has become a necessity for food products, which are sensitive to this type of chemical change. Though widely used synthetic antioxidants are highly effective (*e.g*., BHA, BHT, TBHQ), there is growing consumer demand for natural ingredients application in processed foods. That is why new sources of natural compounds with antioxidant activity have long been sought for. This group includes those plants, which have long been in use due to their positive effect on human body, *e.g*., fruits, vegetables, tea, herbs and spice plants. The high content of antioxidant vitamins such as A, C, E, carotenoids and phenolic compounds in these raw materials enables them to become a source of effective and safe natural antioxidant additives that reduce lipid oxidation [1, 2]. Food phenolics render antioxidant activity mainly due to their role as reducing agents, hydrogen donors, and singlet oxygen quenchers. Some phenolics also have the ability to chelate metal ions, which act as catalysts in oxidation reactions. Flavonoids are natural polyhydroxylated aromatic compounds, that are widely distributed in plants, which have the ability to scavenge free radicals, including hydroxyl, peroxyl, and superoxide radicals and can form complexes with catalytic metal ions rendering them inactive. It has been found that flavonoids can inhibit lipoxygenase and cyclooxygenase enzymes, responsible for development of oxidative rancidity in foods [3]. Aloe is widely used in the cosmetic, pharmaceutical industries and in the food industry, because it contains antioxidants, which may increase the shelf life and nutritional value of food.

## Classification of Antioxidants

Antioxidants are compounds that inhibit or delay onset of oxidation and may be classified as natural or synthetic [1]. The different classes of antioxidants are shown in Table 1.

**Table 1:** Different types of antioxidants [3, 6]

Type of antioxidants	Examples	Function
Free radical scavengers	Synthetic antioxidatns: BHA (Butylated hydroxyanisole) BHT (Butylated hydroxytoluene) TBHQ (tert-Butylhydroquinone) Propyl gallate Natural antioxidants: Tocopherols Aromatic amines Phenolic antioxidants (extracts from aloe, spices and herbs)	Block of the radicals by donating a hydrogen atoms
Oxygen scavengers and reducing agents	Ascorbic acid Erythorbic acid Ascorbates Sulphites, bisulphites Ascorbic palmitate Amino acids	React with oxygen
Chelating agents	Citric acid EDTA (Ethylenediaminetetraacetic acid) Phosphates	Chelate metal ions in their structure in the form of stable complexes to reduce the catalytic oxidation activity

Synthetic antioxidants may cause adverse effects in humans, without any additional nutritional benefits. Due to issues of health safety surrounding food and the growing popularity of food free from synthetic additives, new sources of natural compounds with antioxidant activity have long been sought. Numerous studies confirm the high levels of effectiveness of some plant materials in the reduction of oxidative lipid rancidity. This group includes those plants which have long been used by humans due to their positive effect on the organism – *e.g*., tea, coffee, aloe, herbs and spice plants [4, 5]. The high content of active compounds in these raw materials enables them to become a source of effective and safe natural food additives, hence it seems particularly important to search for appropriate forms of their application and an assessment of their effectiveness in differently processed products. These antioxidative additives, due to long tradition of safe consumption and the content of antioxidative substances, are not only able to reduce the amount of lipid oxidation products, but indeed exhibit this activity in an organism and shape the nutritional value of products. Based on the mechanism of action, antioxidants are categorized as primary antioxidants (radical scavengers), secondary antioxidants (peroxide scavengers), and metal deactivators (complex-forming or chelating agents) [3, 6]. Primary, synthetic antioxidants among others are butylated hydroxyanisole (BHA), butylated hydroxytoluene (BHT), tert-butylhydroquinone (TBHQ), while natural are tocopherols, flavanoids and esters of gallic acid. The secondary antioxidants include peroxide decomposers such as thioethers, methionine, metal chelaters and glutathione peroxidase [4, 6].

## Characteristics of *Aloe vera* L.

### Occurrence and Botanical Characterisation

Aloe is a tropical, drought-resistant succulent. In botany it is known as *Aloe vera* (L.) Webb. (*Aloe barbadensis* Mill.) of the *Liliaceae* family. *Aloe vera* is the most common aloe variety. It is native to the Mediterranean region, the Arabian Peninsula, India, China and Eastern Africa. Wild forms of aloe are common in Cyprus, Malta, Sicily, the Canary Islands, and in India [7]. The biggest *Aloe vera* plantations can be found in the island of Barbados and in the North of the USA. Aloe has branched or unbranched shoots with greyish green sharp-edged coated leaves forming a rosette. The leaves are filled with brown or yellowish milky juice that contains most bioactive compounds.

So far, more than 350 aloe species have been identified. Most of them, *i.e*., 42 species come from Madagascar, 12–15 species from the Arabian Peninsula, 4 from India and the other species come from other tropical countries. About 30 of them have been tested and their therapeutic properties have been confirmed, *e.g*., *Aloe spicata, Aloe perryi* Baker, *Aloe socotńna*, *Aloe africana* Miller, *Aloe chinensis*, *Aloe perfoliata* and *Aloe saponaria*. However, *Aloe barbadensis*, known as *Aloe vera*, *Aloe ferox* (bitter aloe) and *Aloe aborescens* (krantz aloe) are the most common species, typically used for industrial production. Aloe species do not have identical effect on the human organism. Some species are therapeutic, others are toxic or neutral [8].

*Aloe vera* (*Aloe barbadensis* Mill*.*/*Aloe vera* Linn*.*) is the most common aloe variety. It is a short-stemmed perennial, that grows to a height of 60–100 cm. Aloe plants have thick, green or grey and green fleshy, sword-shaped leaves. The leaf edges have triangular thorns at their edges. The flower shoot, which grows in the summer, is built from numerous, pendulous bell-shaped pink and orange flowers. When the plant sheds blossom, it gives fruit in the form of bags [8]. A flesh and pulp obtained from aloe leaves differ in composition and properties. Aloe flesh can be obtained by peeling leaves and then washing and squeezing them carefully. This procedure gives pure flesh without bitter aftertaste or strong laxative properties. Aloe flesh is light green with jelly-like consistency. It is composed of water (96%) and dry matter (4%), which contains protein (6.86%), fat (2.91%), dietary fibre (73.35%), ascorbic acid (0.004%) and ash (16.88%) [9]. Apart from flesh, aloe pulp contains epidermis. It is not washed nor filtered, consequently having strong laxative properties due to the content of aloin.

### Bioactive Components

Aloe contains a large amount of bioactive compounds (Table 2), such as flavonoids, terpenoids, lectins [10, 11], fatty acids, anthraquinones [12], mono- and polysaccharides (pectins, hemicelluloses, glucomannan), tannins, sterols (campesterol, *β*-sitosterol), enzymes, salicylic acid, minerals (calcium, chromium, copper, iron, magnesium, manganese, potassium, phosphorus, sodium and zinc) and vitamins (A, C, E, *β*-carotene, B1, B2, B3, B6, choline, B12, folic acid) [13–15].

**Table 2:** Chemical composition of *Aloe vera* [11, 55, 57, 58]

Compounds	Examples
Non essential and essential amino acids	Alanine, arginine, aspartic acid, glutamic acid, glycine, histidine, hydroxyproline, isoleucine, leucine, lysine, methionine, phenylalanine, proline, threonine, tyrosine, valine
Proteins	Lectins and lectin-like substance
Anthraquinone and anthrone	Aloe-emodin, aloetic acid, anthranol, aloin A and B (barbaloin), isobarbaloin, emodin, ester of cinnamic acid
Enzymes	Alkaline phosphatase, amylase, carboxypeptidase, cyclooxidase, catalase, cyclooxigenase, lipase, oxidase, superoxide dismutase, phosphoenolpyruvate carboxylase, glutathione peroxidase
Hormons	Auxins and gibberellins
Inorganic compound	Calcium, chlorine, chromium, copper, iron, magnesium, manganese, potassium, phosphorous, sodium and zinc
Saccharides	Mannose, glucose, rhamnose
Carbohydrate	Pure mannan, acetylated mannan, acetylated glucomannan, glucogalactomannan, galactogalacturan, arabinogalactan, cellulose, pectic substance, xylan
Vitamines	B1, B2, B6, B12, C, *β*-carotene, folic acid, choline, α-tocopherol
Lipids	Arachidonic acid, *γ*-linolenic acid, sterols (campesterol, cholesterol, *β*-sitosterol,), triglycerides, triterpenoid, gobberellins
Other compounds	Lignin, potassium sorbate, salicylic acid, uric acid

The rich chemical composition of the plant depends on a large number of factors: the type and conditions of cultivation, harvest time, climate, the position of leaves on the stem, aloe species and the method used for harvesting leaves [16]. The optimal time to harvest aloe leaves is after three years of the plant’s growth, because then it has the highest content of polysaccharides (6.55 g/kg) and flavonoids (4.70 g/kg) [8, 17].

### Polysaccharides

There can be different forms of polysaccharides in aloe. Their content depends on the age of the plant (Table 2). Aloe contains a soluble fibre fraction, *i.e*., glucomannan and a hemicellulose component that binds to fibroblast receptors in the cell walls of some plants, enhancing their proliferation. Thus, it accelerates the healing of wounds. Aloe also contains lignins, which aid the absorption of its components through the skin. In consequence, more collagen is produced when aloe is administered locally or externally [18].

Mucopolysaccharides are a special group of polysaccharides in aloe. These chemical organic compounds belong to glycosaminoglycans, which have various functions in the organism [14]. They protect the stomach and duodenum walls from the digestive effect of pepsin. They activate the protective barrier of the mucosa through the stimulation of mucus secretion and reduce susceptibility to allergies and irritations. They have positive influence on the flow of blood and lymph, in consequence preventing the formation of cellulite. Mucopolysaccharides moisturise the skin by water retention [14, 19]. Hyaluronic acid, heparin and acemannan are the main mucopolysaccharides found in aloe, however among them acemannan is the most abundant. It has a long carbon chain, which is mainly composed of uronic acids and amino sugars. Acemannan has bactericidal, virucidal and fungicidal properties. It is also responsible for the immune reactions of the organism and one of the strongest immunomodulators of plant origin. Acemannan activates macrophages that bind and destroy microorganisms. It accumulates in cell membranes, where it makes a specific protective barrier and consequently tightens cell walls. As a result, it inhibits the absorption of toxins from the intestine into the cardiovascular system. It also aids the regeneration of natural bacterial flora. This polysaccharide ensures the normal flow of blood and lymph and gas exchange in the alveoli. It maintains the optimal moisture in cartilages and facilitates the absorption of nutrients and water in alimentary tract [20]. The human organism produces acemannan until the age of adolescence. Different factors such as stress reduce its content in the organism. The acemannan deficit is manifested by swellings, lymphostasis, digestion problems, joint and nerve root pains and various infections. It is also manifested by typical symptoms of poisoning and hypoxia [20]. Therefore, aloe consumption is a method of acemannan supplementation to improve the functioning of the organism.

### Glycoproteins

Glycoproteins are natural polymers that combine proteins, carbohydrates and 16 amino acids. There are considerable amounts of glutamic and aspartic acids [21]. Glycoproteins are responsible for the identification of antibodies and prevent the breakdown of peptide bonds (proteolysis).

### Lectins

Lectins (aloctin A and B) are a group of glycoproteins, which is characteristic of aloe. They differ in the connection between oligosaccharide groups and the polypeptide chain. Aloctin A connects them with the O-glycosidic bond through serine or threonine rest, but aloctin B connects them with the N-glycosidic bond through asparagine rest. Lectins mainly play the mitogenic and immunochemical role. This means that they stimulate cell divisions and affect the growth of the number of B- and T-lymphocytes. The mechanism of action is based on the activation of cell blastic transformation, where the cell transformation from phase G0 (resting phase) to phase G1 (interphase) or synthesis is induced. Mitotic divisions are stimulated in consecutive processes [8].

Aloctins destroy cancer cells. They agglutinate carcinogenic cells by binding the polysaccharide fragments of their membranes with the active centre of aloctin [22].

### Anthraquinones

Anthraquinones are anthracene derivatives of the quinone group. They are mostly known as components of strong or mild laxatives. According to recent reports, new origin-dependent properties of anthraquinones were discovered. They were proved to exhibit antioxidative, antiviral and cytotoxic effect on squamous cell lung cancer as well as bacteriostatic effect on *Streptococcus viridans*. There are also investigations to confirm the health-beneficial effect of anthraquinones on patients with malaria as well as viral and fungal infections. In most studies, experiments are at the stage of assessment of the functional properties of anthraquinones [23]. Aloe-emodin and aloin are the main anthraquinones in aloe [24]. Aloe-emodin is a compound with the primary alcohol group, mainly found in aloe juice. It is offered commercially as a powder and it is mostly a laxative. After consumption, anthra-compounds are partially absorbed in the small intestine, whereas glycoside bonds reach the large intestine, where they are hydrolysed into aglycones. Oxidation and reduction also take place. In consequence, anthranol and anthrones are produced directly irritating the intestinal mucosa. There is increased secretion from the large intestine, peristalsis is stimulated and water absorption in the intestine is inhibited. Aloe-emodin also exhibits the antioxidative effect. It has been confirmed to inhibit the oxidation of linolenic acid by 78%. Its effect is caused by very strong reducing properties and the capacity to scavenge hydroxyl free radicals [25]. It is also cytotoxic to CH27 strain cells of squamous cell lung cancer in humans. The mechanism of action is based on increased activity of cytochrome C, which is a transporter in mitochondria. It migrates to the cytoplasmic space and binds with adaptor proteins that become active only after association with another substance. It activates caspase enzymes, which cause cell apoptosis.

Aloin (barbaloin) is another representative of anthraquinones. It is aloe emodin anthrone C-glucoside, It exhibiting very similar properties to aloe-emodin. Both substances are mainly laxatives. Apart from that, aloin inhibits lipid peroxidation in the cerebral cortex by inactivation of Fe(II)-dependent ascorbate.

### Enzymes

Aloe contains numerous enzymes such as: alkaline phosphatase, amylase, bradykinase, catalase, lipase, protease, creatine phosphokinase, carboxypeptidase, cellulase, and oxidase [8]. Superoxide dismutase is the most important and active enzyme found in aloe [19].

### Phenolic Compounds

There is diversified content of phenolic compounds in individual morphological parts of aloe (Table 3). The content of phenolic compounds in aloe’s leaf epidermis is higher than in flowers. Leaves contain the most catechin (95.0 mg/100 g), whereas flowers contain most genistic acids (101.0 mg/100 g) [26].

**Table 3:** The content of selected polyphenols in aloe extracts [26]

Phenolic compound	Leaf skin^*^	Flowers^*^
Catechin	95.0	7.6
Sinapic acid	54.0	15.0
Quercetin	34.4	nd
Quercitrin	23.0	31.9
Rutin	22.3	11.6
Miricetin	19.6	1.8
Epicatechin	16.2	58.0
Gentisic acid	6.0	101.0

^*^mg *per* 100 g of freeze-dried aloe material

nd – not detected

The total content of phenolic compounds in aloe’s leaf epidermis and flowers amounts to 307.5 and 274.5 mg *per* 100 g of lyophilised material, respectively [26].

### Antioxidant Activity

The *in vitro* antioxidant activity of *A. arborescens* [27], *A. ferox* [28–30], *Aloe greatheadii* var. *Davyana* [31], *A. harlana* [32], *A. saponaria* [33], *A. marlothii*, and *A. melanacantha* [34] leaf extracts were reported in the literature. Sazhina et al. [35] reported that leaf extracts from 15 *Aloe* species exhibited high antioxidant activity. *A. ferox* antioxidant capacity was determined using ORAC and FRAP analyses [28]. Authors reported that due to the FRAP analysis being an indication of the ferric ion reducing power of a compound or mixture and the ORAC analysis indicating the ability of a compound or mixture to scavenge free radicals, the various individual polyphenol components of the mixture may have stronger free radical scavenging abilities than reducing power, or vice versa, depending on their chemical structures. As a result, it can be used in alleviating symptoms or preventing oxidative stress-related diseases. Wintola and Afolyan [30] noted than the free radical scavenging activity of the methanol, acetone and ethanol extracts of *Aloe ferox* Mill at a concentration of 0.5 mg/mL, showed higher inhibition against ABTS, hydrogen peroxide and nitric oxide radicals. Whereas, scavenging activity of the extracts against DPPH and lipid peroxidation were was observed at a concentration of 0.016 and 0.118 mg/mL, respectively in comparison to BHT, gallic acid and rutin. The ferric reducing potential of the extracts was concentration dependent and significantly different from that of vitamin C and BHT. Aloeresins in *A. ferox* displayed strong antioxidant activity [36]. Also, *A. ferox* leaves methanol extract showed good DPPH scavenging activity [37]. The total flavonoid contents and the antioxidant capacities of *A. greatheadii* lyophilized leaf gel, as measured by ORAC, were however higher comparatively, which may be indicative of the types of polyphenols in *A. greatheadii* having stronger scavenging ability than ferric ion reducing potential [31] (Table 4). The DPPH radical-scavenging activities of freeze-dried whole leaf, freeze-dried leaf skin, and boiled leaf skin were 48.2, 35.0, and 61.6 mM Trolox equivalent/g, respectively. DPPH radical-scavenging activity was increased by boiling of freeze-dried leaf skin [27]. Likewise, Ray et al. [38] observed that aloe gel and the methanol extract of aloe gel exhibited low DPPH neutralisation capacity. However, these preparations exhibited much higher metal chelation capacity than the aqueous extract of aloe. Aloe gel inhibited the generation of DPPH radical in a dose-dependent manner and its IC_50_ value was found to be 572.14 μg/mL, which is defined as the concentration of substrate that causes 50% loss of the DPPH activity (colour). A lower value of IC_50_ indicates the greater antioxidant activity of a test substance. Aloe scavenged the ABTS in a dose dependent manner and its IC_50_ value was found to be 105.26 μg/mL.

**Table 4:** Antioxidant activity of various forms of aloe

Sample	Methods	Antioxidant activity	References
Gel extract	DPPH radical scavenging (%)	11.93	[42]
	Ferric reducing power (μM Fe(II)/kg)	59.12	
Ethanol extract of gel	DPPH radical scavenging (%)	6.56	
	Ferric reducing power (μM Fe(II)/kg)	26.51	
Ethanol extract of skin	DPPH radical scavenging (%)	85.01	
	Ferric reducing power (μM Fe(II)/kg)	185.98	
Methanol extract of skin	DPPH radical scavenging (%)	58.80	[26]
	Ferric reducing power (mM of Fe(III) reduced to Fe(II))	2.40	
Methanol extract of flowers	DPPH radical scavenging (%)	53.00	
	Ferric reducing power (mM of Fe(III) reduced to Fe(II))	1.70	
Gel	DPPH radical scavenging (%)	13.52	[59]
	Hydroxyl radical scavenging (%)	11.74	
	Superoxide radical scavenging (%)	53.86	
	Metal chelating activity (%)	81.27	
Methanol extract of gel	DPPH radical scavenging (%)	10.24	[38]
	Hydroxyl radical scavenging (%)	48.01	
	Superoxide radical scavenging (%)	31.72	
	Metal chelating activity (%)	48.02	
Lyophilized leaf gel	Oxygen radical absorbance capacity (μM of TE^a^/g d.m.)	59.00	[31]
	Ferric reducing power (μM/g d.m)	2.63	
Aqueous ethanol leaf gel extracts	Oxygen radical absorbance capacity (μM of TE/g d.m.)	83.00	
	Ferric reducing power (μM/g d.m)	8.98	
Leaf extract	DPPH radical scavenging (IC_50_^b^ mg/mL)	Methanol (0.086) > ethanol (0.288) = acetone (0.288) > aqueous extract (0.517)	[30]
	ABTS cationic radicals scavenging (IC_50_ mg/mL)	Methanol (0.02) > acetone (0.033) > ethanol (0.062) > aqueous extracts (0.173)	
	Ferric reducing power (absorbance)	Ethanol > acetone > methanol > aqueous extracts	
	Nitric oxide scavenging (IC_50_ mg/mL)	Methanol (0.023) > ethanol (0.024) > aqueous (0.074) > acetone extracts (0.077)	
	Hydrogen peroxide scavenging (%)	Acetone < ethanol < methanol aqueous extract	
	Lipid peroxidation (TBARS^c^) (IC_50_ mg/mL)	Methanol (0.930) > ethanol (1.270) > acetone (1.492) aqueous extract (1.837)	
Freeze-dried whole leaf	DPPH-HPLC method radical-scavenging (mM of TE/g)	48.20	[27]
Freeze-dried leaf skin		35.00	
Boiled leaf skin		61.60	
Lyophilized leaf gel	Oxygen radical absorbance capacity (μM of TE/g d.m.)	53.00	[28]
	Ferric reducing power (μM/g d.m)	4.90	
Ethanol leaf gel extracts	Oxygen radical absorbance capacity (μM of TE/g d.m.)	136.00	
	Ferric reducing power (μM/g d.m)	19.00	
Methanol extract of aloe leaves	DPPH radical scavenging (EC_50_^d^) (μg/mL)	10.45	[29]
	Ferric reducing power (absorbance)	~ 0.50	
Latex from the leaves of aloe	DPPH radical scavenging (IC_50_) (μg/mL)	14.21	[32]
	2-Deoxyribose degradation assay (IC_50_) (μg/mL)	17.24	
Ethanol extract of leaf of aloe leaves	DPPH radical scavenging (IC_50_) (μg/mL)	73.00	[33]
	Assay for inhibition of xanthine oxidase activity (IC_50_) (μg/mL)	85.00	
Aloe gel	DPPH radical scavenging (IC_50_) (μg/mL)	572.14	[39]
	ABTS cationic radicals scavenging (IC_50_) (μg/mL)	105.26	
	Nitric oxide scavenging (IC_50_) (μg/mL)	46.36	
Ethanol extracts of leaf *Aloe barbadensis*	DPPH radical scavenging (μM of TE)	108.00	[40]
	Oxygen radical absorbance capacity (μM of TE)	1281.00	
Ethanol extracts of leaf *Aloe arborescens*	DPPH radical scavenging (μM of TE)	71.00	
	Oxygen radical absorbance capacity (μM of TE)	2671.00	
Water extract of aloe	DPPH radical scavenging (mg of TE/g d.m.)	8.87	[43]
	ABTS cationic radicals scavenging (mg of TE/g d.m.)	0.87	
	Metal chelating activity (mg of EDTA^e^/g d.m.)	8.76	
	Emulsion system (Wo)^f^	0.96	
Ethanol extract of flowers	DPPH radical scavenging (IC_50_ mg/mL)	0.25	[60]
	ABTS cationic radicals scavenging (IC_50_ mg/mL)	0.30	
	Ferric reducing power (EC_50_ mg/mL)	2.10	
	Nitrite scavenging (IC_50_ mg/mL)	0.92	
Methanol extracts of leaf	DPPH radical scavenging (%)	56.75–80.20	[44]
	Metal chelating activity (%)	55.00–80.00	
	Hydrogen peroxide scavenging (%)	58.54–81.10	
	Ferric reducing power (absorbance)	0.6–0.8	
	*β* carotene-linoleic assay (%)	59.60–74.40	
Aloe barbadensis leaves	Ferric reducing power (absorbance)	Extraction by shaker: Absolute methanol (2.01), Aqueous (80%) methanol (2.81), Absolute ethanol (1.56), Aqueous (80%) ethanol (2.16)	[45]
		Extraction by reflux: Absolute methanol (2.18), Aqueous (80%) methanol (2.96), Absolute ethanol (1.72), Aqueous (80%) ethanol (1.88)	
	DPPH radical scavenging (%)	Extraction by shaker: Absolute methanol (73.7), Aqueous (80%) methanol (80.1), Absolute ethanol (67.2), Aqueous (80%) ethanol (70.7)	
		Extraction by reflux: Absolute methanol (72.9), Aqueous (80%) methanol (77.6), Absolute ethanol (68.0), Aqueous (80%) ethanol (71.9)	
	Inhibition of linoleic acid peroxidation (%)	Extraction by shaker: Absolute methanol (66.2), Aqueous (80%) methanol (68.3), Absolute ethanol (63.7), Aqueous (80%) ethanol (65.9)	
		Extraction by reflux: Absolute methanol (64.3), Aqueous (80%) methanol (67.9), Absolute ethanol (66.2), Aqueous (80%) ethanol (67.3)	

^a^TE – Trolox equivalents

^b^IC_50_ – The concentration at which 50% is inhibited

^c^TBARS – Thiobarbituric acid reactive substances

^d^EC_50_ – Effective concentration at which the absorbance is 0.5

^e^EDTA – Ethylenediaminetetraacetic acid equivalents

^f^Wo – Antioxidant efficiency (Wo > 0 antioxidative properties, Wo < 0 prooxidative properties of the additive)

The gel resulted in inhibition of NO generation in vitro in a concentration-dependent manner and its IC_50_ value was calculated as 46.3 μg/mL [39]. The IC_50_ of the latex from the leaves of aloe in DPPH assay was found to be 14.21 μg/mL, while that of ascorbic acid was 4.76 μg/mL. Isolated compounds exhibited a free radical scavenging property depending on the concentration. The degradation of deoxyribose to TBARS by hydroxyl radical generated from the Fe(III)–ascorbate–EDTA–H_2_O_2_ system was markedly decreased by the latex in a concentration dependent manner. Its IC_50_ value was found to be 17.24 μg/mL, which was higher than that of the standard BHT (4.63 μg/mL) [32]. To investigate the antioxidant capacity of *A. saponaria* Haw., Yoo et al. [33] measured the antioxidant activity of an ethanol extract from the leaf of aloe using xanthine-xanthine oxidase (XO) assay. The assay was carried out at ten different concentrations ranging from 1 to 500 μg/mL. The ethanol fraction from *A. saponaria* Haw. displayed significant dose-dependent inhibition. The ethanol fraction was found to have an IC_50_ value of 85 μg/mL. In order to confirm the scavenging effect of *A. saponaria* Haw., DPPH assay was conducted and concentration-dependent DPPH radical scavenging activity. The ethanol fractions highly scavenged the radical generation with an IC_50_ value of 73 μg/ml [33]. 5-Methylchromones aloesin, aloeresin A, and aloesone, which are compounds present in *A. barbadensis* and *A. arborescens*, exhibited the most radical scavenging activity by DPPH and ORAC assays [40]. Hassanpour [41] noted than raspberry fruits treated with *Aloe vera* gel maintained higher levels of antioxidant capacity, total phenol, total anthocyanin and antioxidant enzymes during storage periods. Moniruzzaman et al. [42] and Lόpez et al. [26] observed that the aloe epidermis extract exhibited greater activity than aloe gel. They found that alcohol extracts of aloe epidermis exhibited greater DPPH radical-scavenging activity and greater capacity to reduce Fe(III) to Fe(II) than aloe flower extracts. The capacity of whole leaf extracts to scavenge the DPPH radical and ABTS cationic radical depended on the extractant applied. Methanol extracts were characterised by the highest activity. The gel extract exhibited greater radical-scavenging capacity than the whole leaf’s extract. The extracts produced by means of hexane were characterised by the lowest activity (Table 4). The research conducted by Hęś et al. [43] showed diverse mechanisms of action for antioxidants in the aloe extract. The research results showed the high antioxidative potential of aloe aqueous solution in the emulsion system. The protective effect of linoleic acid was similar to the effect of BHT. The extract exhibited low iron ion fixing activity as well as the activity in fixing DPPH stable-free radicals and ABTS cationic radicals (Table 4). These differences in the activity of aloe preparations may have been caused by different methods of extraction of phenolic compounds, the solvent used for extraction and the morphological part of the plant (aloe’s leaf epidermis and gel). The content of antioxidants in aloe also depends on numerous factors such as the type and conditions of cultivation, harvest time, climate, the position of leaves on the stem, aloe species and the method used for harvesting leaves [16, 44]. Moniruzzaman et al. [42] proved that the content of flavonoids and the total amount of phenolic compounds in ethanol aloe extracts were strongly correlated with their DPPH radical scavenging capacity and ferric reducing power. The available literature does not provide many studies on the antioxidative potential of aloe in model systems, especially in emulsified fat systems and mass-fat systems.

Kumar et al. [44] observed that different agro-climatic conditions have effects on the phytochemicals, total phenolic content and antioxidant potential of the *A. vera* plant. The authors studied the crude methanolic extracts of *A. vera* from the different states of India for presence of various phytochemicals, total phenolic content and *in vitro* antioxidant activity. A significant positive correlation was detected between total phenolic content and antioxidant activity of different accessions. Extracts of highland and semi-arid zones possessed maximum antioxidant potential. Accessions from tropical zones showed the least antioxidant activity in all assays. Sultana et al. [45] checked for antioxidant activity of *Aloe barbadensis* leaf extracts obtained using four extracting solvents and two unique extraction techniques. They reported that the extracts had good DPPH scavenging properties. The ethanolic extracts of leaves of *A. barbadensis* prepared by the reflux technique showed better scavenging activity as compared with those prepared by the shaking technique. The aqueous organic solvent extracts of *Aloe barbadensis* exhibit greater reducing power than absolute solvents (Table 4). In an *in vivo* studiy, Golestan et al. [46] showed that dietary *Aloe vera* have adverse effects on antioxidant defense system in rainbow trout (*O. mykiss*). A total number of 480 *O. mykiss* were randomized into one control and three experimental groups where aloe was incorporated in their diet at 0.5, 1 and 2 g/kg. Trial conducted lasted eight weeks. Then biometry and blood sampling were identified. Plasma malondialdehyde, ferric reducing ability of plasma and growth index were estimated at the end of study. The results showed that *Aloe vera* extract did not affect growth indices. Malondialdehyde was increased in the experimental group compared to the control but ferric reducing ability of plasma showed a decrease in experimental groups (*p* < 0.05) compared to the control group. It has also been proved that in the occurrence of copper ions in the aqueous extract of *Aloe vera* the DNA is degraded and there is a reduction of Cu(II) to Cu(I) along with generation of reactive oxygen species such as superoxide anion and hydroxyl radicals in a dose-dependant manner. This observation demonstrates the pro-oxidant property in addition to its antioxidant property. Investigations of the antioxidant potential of a polysaccharide isolated from *Aloe vera* gel showed that it had a protective effect against dihydrochloride induced oxidative stress and cell’s death in kidney epithelial cells [47]. Antioxidant compounds present in *A. saponaria* gel exerted antinociceptive and anti-inflammatory effects by the topical treatment of an ultraviolet B-induced sunburn model [48]. Nwajo [49] performed the treatment of diabetic rats with the aid of leaves of *Aloe barbadensis* (150 mg/kg) which increases the antioxidant enzymes like superoxide dismutase (SOD) activities, and significantly reduced the lipid peroxidation products. They concluded that augmented blood sugar leads to amplified oxidative stress and it was found that extract of *Aloe barbadensis* leaves possesses good antioxidant activities. It was also observed that there is a noteworthy rise in reduced glutathione, superoxide dismutase, catalase, glutathione peroxidase and glutathione-S-transferase in the liver and kidney of the treated rat [50]. Esteban et al. [51] has analysed the basic peroxidase in the commercially available aloe gel as well as in the *Aloe vera* plant and it has been found to occur in the vascular system of the internal aqueous leaf parenchyma. It was determined that peroxidase enzyme in skin surface possibly can scavenge H_2_O_2_. This illuminates that the *Aloe vera* plant has been constituted with agreeable antioxidant properties; moreover, this can serve as an evidence for taking this plant-based products further to the treatment of numerous diseases.

### Health-Promoting Effect and Health Safety

The bioactive components of aloe have anti-inflammatory effect and aid the treatment of gastrointestinal diseases, *i.e*., inflammations, gastric, duodenal and intestinal ulcers. They aid lipid and carbohydrate metabolism, which helps to maintain normal blood sugar and cholesterol levels as well as normal body weight. Due to aloin, the daily intake of aloe juice should not exceed 30–40 mL, because excessive consumption may not only have a strong laxative effect but also toxic effects. When aloe is applied externally, it helps to regenerate burnt or frostbitten skin [12, 13, 52, 53].

**Fig. 1:**
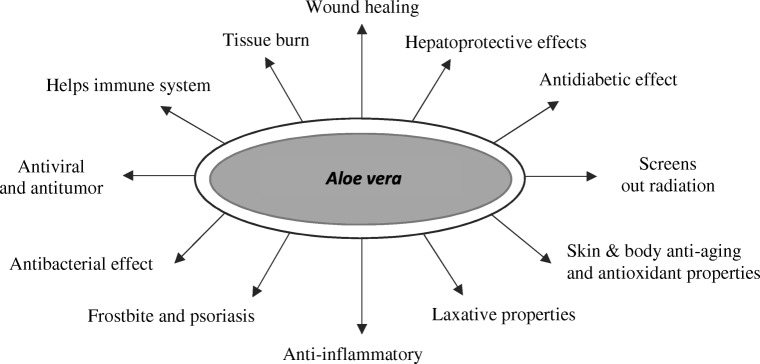
Medicinal uses of *Aloe vera* plants [45, 55, 56]

Figure 1 shows the medical use of *Aloe vera* plants. It should be noted that hydroxyanthracene derivatives occurring naturally in aloe have a strong purgative action, which may pose a threat to the health of humans.

As anthraquinones are commonly used as active substances in slimming and metabolism-improving preparations, they are often abused. Long-term use of anthraquinones may result in intestine inflammation, it may prolong menstrual bleeding and cause difficulties in the absorption of nutrients [24]. Due to aloin, the daily intake of aloe juice should not exceed 30–40 mL, because excessive consumption may not only have a strong laxative effect but also toxic effects [54]. Apart from that, aloin inhibits lipid peroxidation in the cerebral cortex by inactivation of Fe(II)-dependent ascorbate.

## Conclusions

The development of medical sciences, dietetics and nutrigenomics confirmed the correlation between the food consumed and human health. Consumers have increasingly favoured food products that contain natural ingredients due to concerns over adverse health effects of synthetic raw materials particularly some synthetic antioxidants. During the storage and processing of foods, lipids (especially those rich in polyunsaturated fatty acid residues) are oxidized. Oxidation processes are major causes of deteriorating quality. They are responsible for the degradation of aroma, taste, texture and consistency, as well as decreases of nutritive value. In addition, lipid oxidation possess health risks due to peroxides that can cause oxidation damage in living tissues. Products from lipid oxidation such as lipid peroxides and aldehydes can also induce mutagenesis and carcinogenesis. Therefore, the priority is to maximize the reduction of the oxidative metabolism of lipids. The use of plant extracts as antioxidants is gaining popularity and is widely accepted by consumers, as food additives derived from natural raw materials are known to be safe. In conclusion, polyphenols derived from aloe may exhibit a number of properties in various modelling systems. Interests in food antioxidants will continue to increase as well as research and technology that will develop better ways of growing aloe containing higher amounts of antioxidants. Enrichment of aloe food products with polyphenols can beneficially influence their oxidative stability and thanks to additional introduction to the human organism, it may contribute to a decline in the incidence of degenerative diseases.

## Abbreviations

ABTS: 2, 2-azinobis (3-ethylbenzothiazoline- 6-sulfonic acid)
BCFA: branched-chain fatty acids
BHA: butylated hydroxyanisole
BHT: butylated hydroxytoluene
cAMP: 3′,5′-cyclic adenosine monophosphate
DPPH: 2,2-diphenyl-1-picrylhydrazyl
EC_50_: effective concentration at which the absorbance is 0.5
EDTA: ethylenediaminetetraacetic acid
FISH: fluorescence *in situ* hybridization
FOS: fructooligosaccharides
FRAP: ferric reducing antioxidant power
HDL: high-density lipoprotein
IC_50_: inhibitory concentrations 50% of cells
LDL: low-density lipoprotein
nd: not detected
ORAC: oxygen radical absorbance capacity
qPCR: quantitative polimerase chain reaction
SCFA: short chain fatty acids
SOD: superoxide dismutase
TBARS: thiobarbituric acid reactive substances
TBHQ: tert-butylhydroquinone
TE: trolox equivalents
Wo: antioxidant efficiency
